# Supplementary material for: Tuning the zeolite acidity enables selectivity control by suppressing ketene formation in lignin catalytic pyrolysis
Source: Nat Commun. 2023 Jul 27;14:4512. doi: 10.1038/s41467-023-40179-z (PMC10374901; doi:10.1038/s41467-023-40179-z)
Supplement: Supplementary file 1 — Supplementary Information [file 41467_2023_40179_MOESM1_ESM.pdf]

Supplementary Information

Tuning the Zeolite Acidity Enables Selectivity Control by Suppressing Ketene Formation in Lignin Catalytic Pyrolysis

Pan et al.

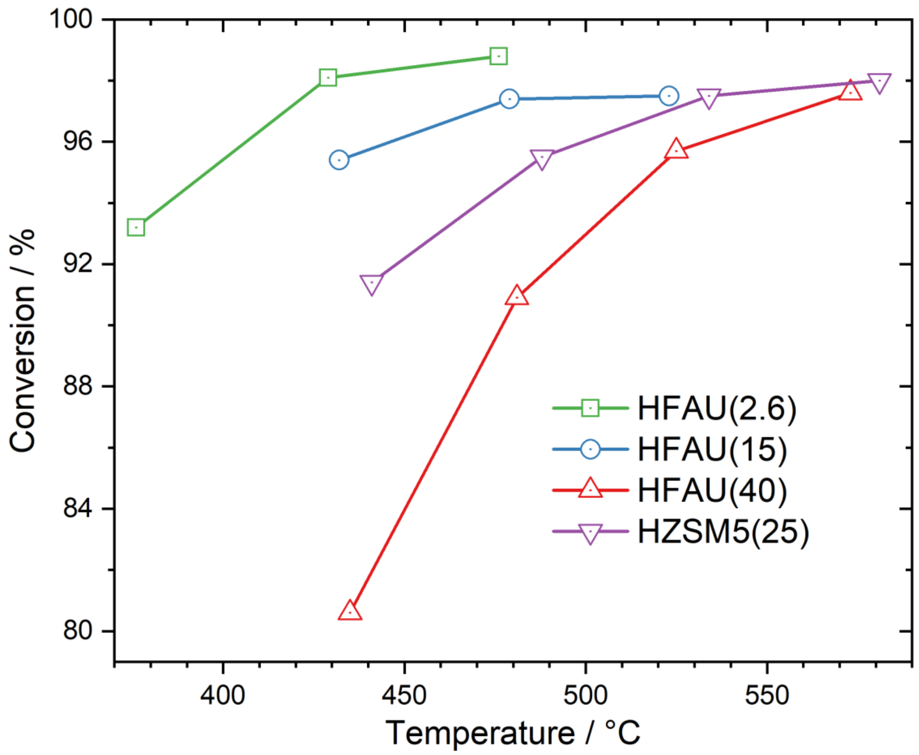

Supplementary Fig. 1 | Temperature-dependent conversion of guaiacol over zeolite catalysts, calculated by comparing guaiacol signal without catalyst at room temperature and with catalyst at reaction temperature. The guaiacol concentration in Ar was 0.01%.

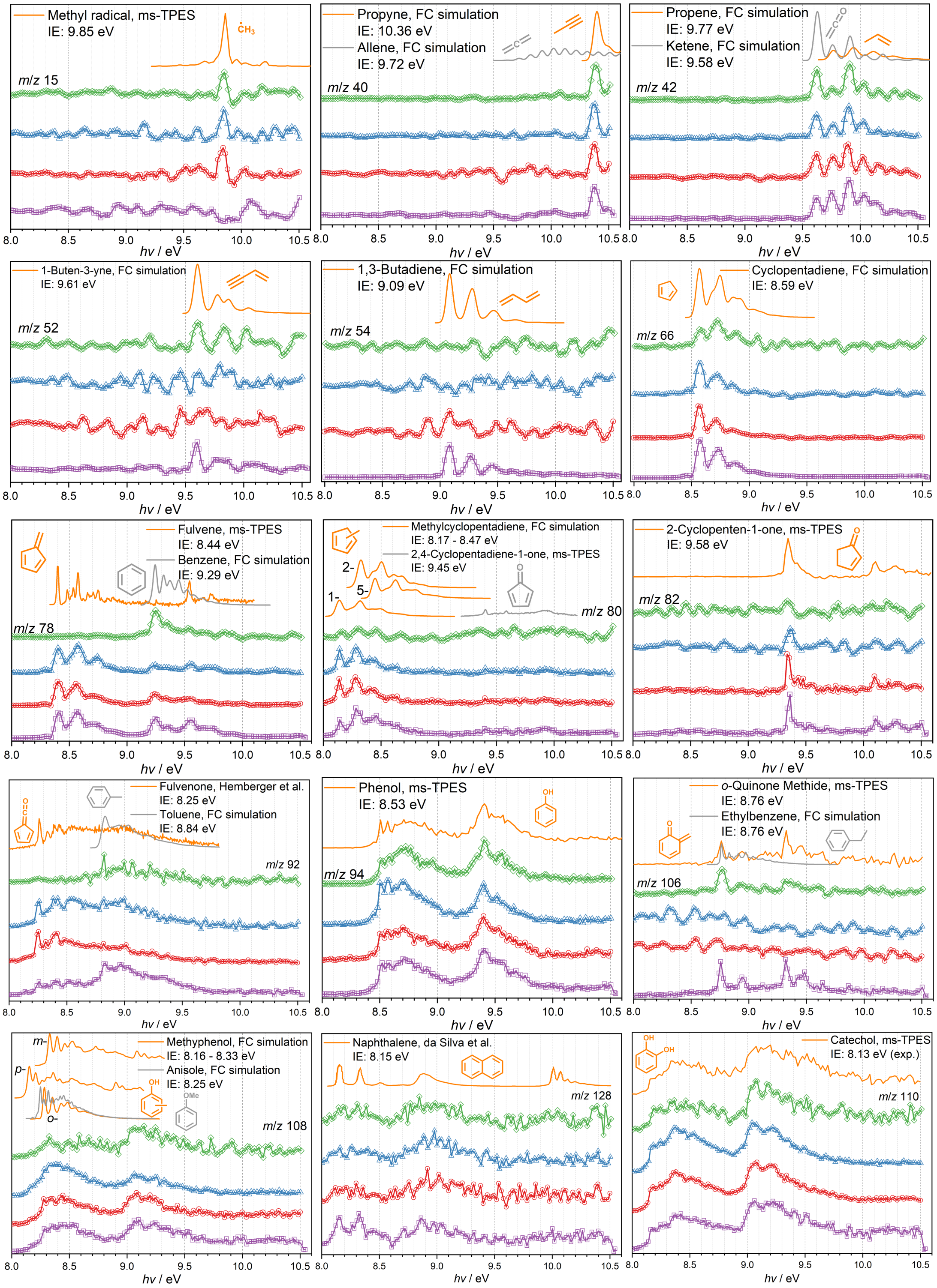

Supplementary Fig. 2 | Photoion mass-selected threshold photoelectron spectra (ms-TPES) along with Franck–Condon (FC) simulations or reference spectra. Purple, red, blue, and green traces correspond to the effluent over HZSM5(25), HFAU(40), HFAU(15), and HFAU(2.6) catalysts, respectively. Orange and grey traces represent FC simulations and literature reference spectra for assignment.[1,2]

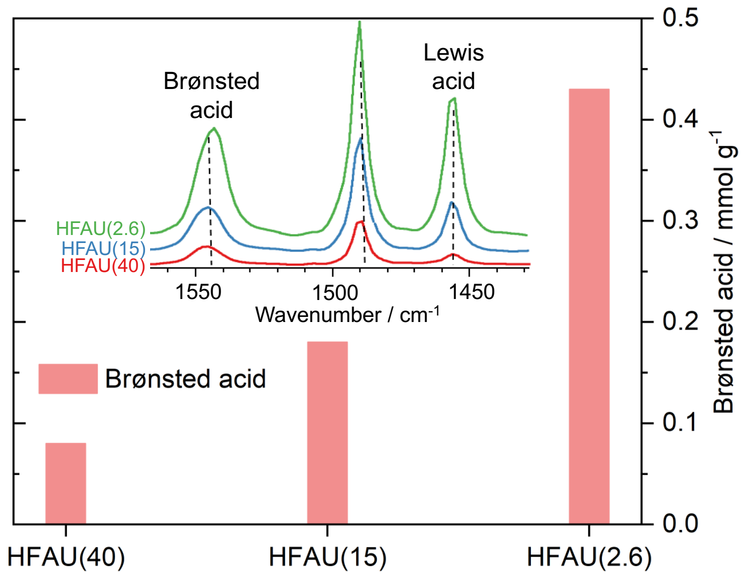

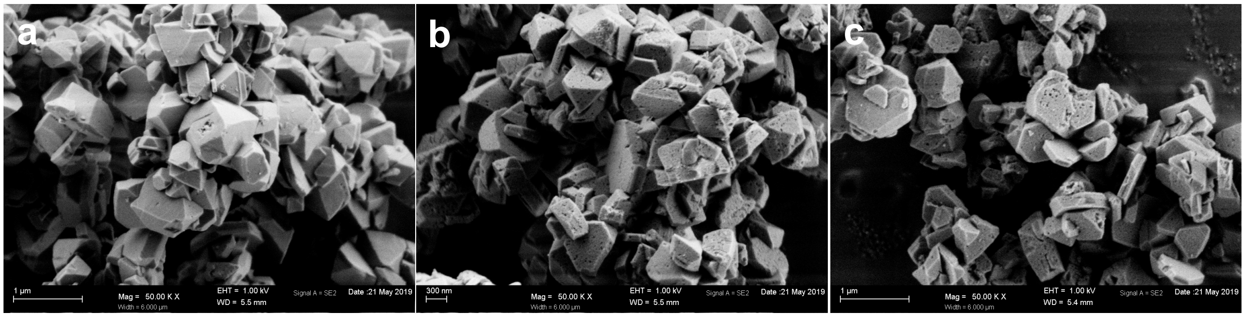

Supplementary Fig. 3 | Top: Fourier-transform infrared (FTIR) spectroscopy results of pyridine adsorption on HFAU. Brønsted acid sites are quantified based on the 1544 cm^{-1} band. The amount of Brønsted acid sites increases when lowering the Si/Al ratio and reaches the maximum on HFAU(2.6) at 0.43 mmol/g, which is ca. 5 times more than in HFAU(40). Bottom: scanning electron microscope images of HFAU catalysts. a) HFAU(2.6), b) HFAU(15), c) HFAU(40).

Supplementary Table 1 | Textural properties of HFAU catalysts

	V_{micro} (cm^3/g)	V_{meso} (cm^3/g)	V_{total} (cm^3/g)	Ref.
HFAU(2.6)	0.27	0.02	0.30	3
HFAU(15)	0.30	0.13	0.44	
HFAU(40)	0.21	0.26	0.46	4

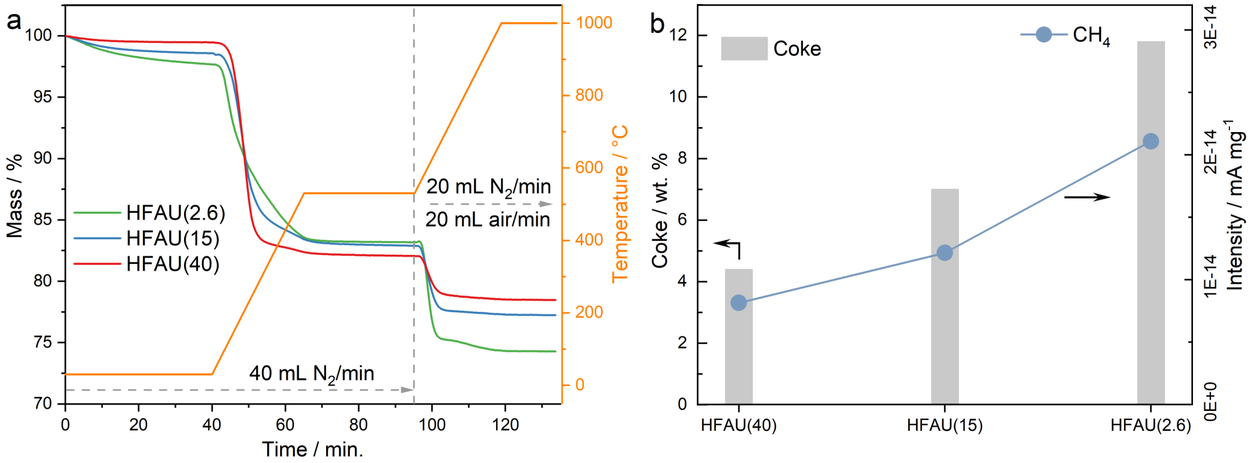

Supplementary Fig. 4 | Guaiacol pyrolysis studied by thermogravimetric analysis coupled with mass spectrometry (TGA/MS). **a**, Mass loss as a function of temperature. **b**, Methane and coke yields as function of the Si/Al ratio. To reduce the influence of the slow heating rate on the results, the mass spectrometer continuously collected data (all signals were summed up) during the two thermal steps (i.e., for 55 min). Most of this time, the system was at a temperature around which all the catalysts tested presented a similar conversion (Supplementary Fig. 1)

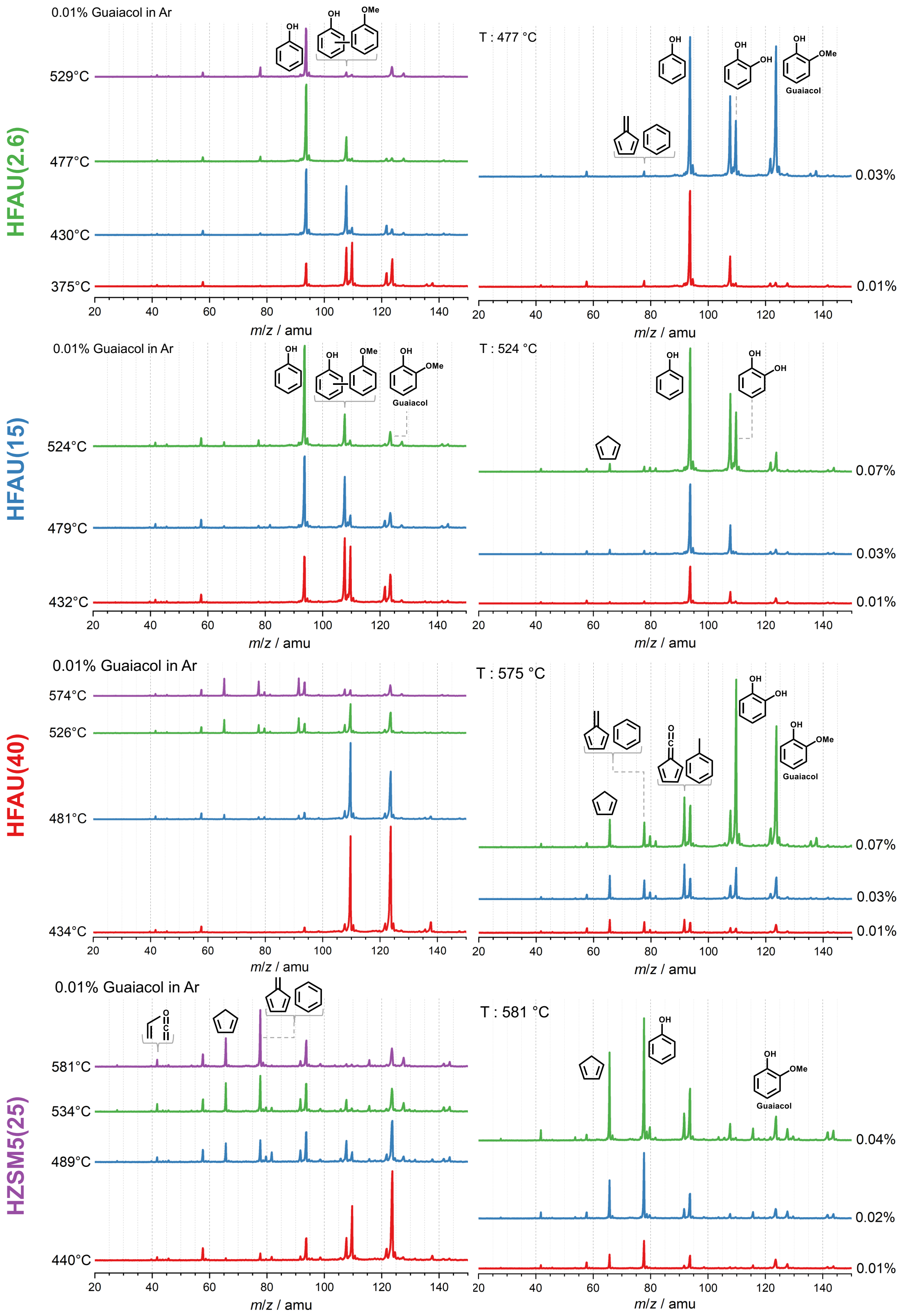

Supplementary Fig. 5 | Temperature- and concentration-dependent photoionization mass spectra of guaiacol catalytic fast pyrolysis (CFP) products over different catalysts, recorded at a photon energy of 10.5 eV.

Supplementary Note 1: Signal quantification

To quantify intermediates and products, their mass spectral peak integral is first normalized to that of acetone at *m*/*z* 58, which acts as a constant internal calibrant background peak in the ionization chamber. The mass discrimination factor was found to be close to unity.[5] After normalization, the signal is divided by the molecule and photon energy dependent photoionization cross section (PICS):

$$M_{\mathrm{A}} = \frac{S_{\mathrm{A}}}{\sigma_{\mathrm{A}}}$$

where M_{A} and S_{A} represents the normalized and spectral signal for species A respectively, and σ_{A} stands for the PICS of A at the detection photon energy. The mole fraction of A, m_{A}, is obtained as:

$$m_{\mathrm{A}} = \frac{M_{\mathrm{A}}}{M_{\mathrm{A}} + M_{\mathrm{B}} + \cdots + M_{\mathrm{N}}}$$

Additionally, the isomer fractions of the *m*/*z* 78 and 92 peaks were estimated based on the intensity of the first vibrational peak in the ms-TPES.

Supplementary Table 2 | Photoionization cross sections

	Formula	*m*/*z*	PICS (Mb) @ 10.5 eV	Ref.
Cyclopentadiene	C_5H_6	66	31	[6]
Benzene	C_6H_6	78	30	[7]
Fulvene	C_6H_6	78	33.2	[8,9]
Fulvenone	C_6H_4O	92	18.8	[5]
Toluene	C_7H_8	92	31.2	[10]
Phenol	C_6H_6O	94	33.4	[11]
Methylphenol	C_7H_8O	108	40	[12]
Anisole	C_7H_8O	108	40	Estimated[a]
Catechol	C_6H_6O	110	36.8	Estimated[b]

[a] $\mathrm{PICS_{anisole}} \cong \mathrm{PICS_{Methylphenol}}$, based on structural similarity of anisole and methylphenol.

[b] $\mathrm{PICS_{catechol}} \cong \mathrm{PICS_{phenol}} + \mathrm{PICS_{hydroxyl\ group}} \cong \mathrm{PICS_{phenol}} + (\mathrm{PICS_{phenol}} - \mathrm{PICS_{benzene}})$.

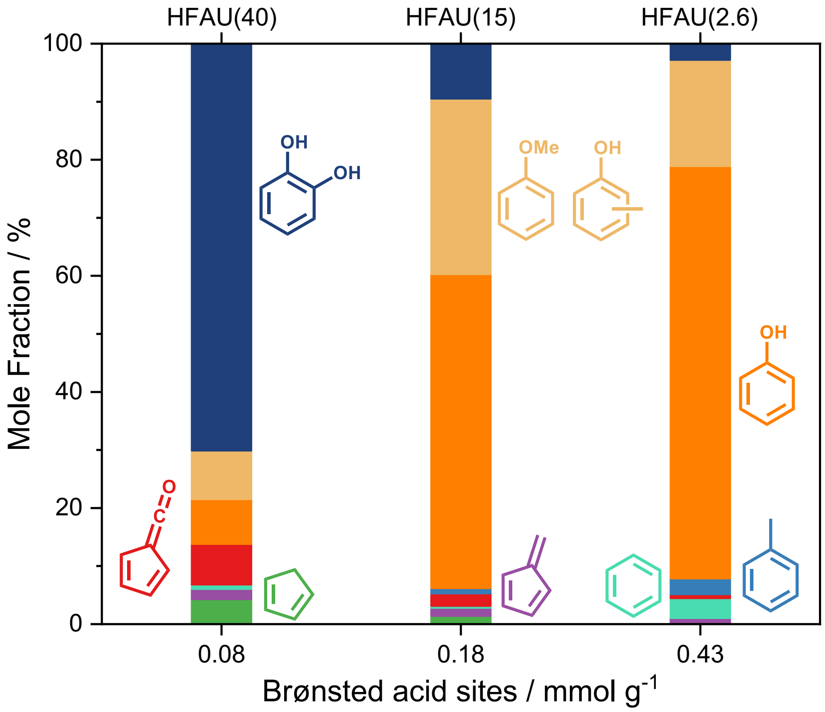

Supplementary Fig. 6 | Mole fraction of the main products and intermediates as a function of the density of Brønsted acid sites in HFAU during guaiacol CFP at ca. 480 °C.

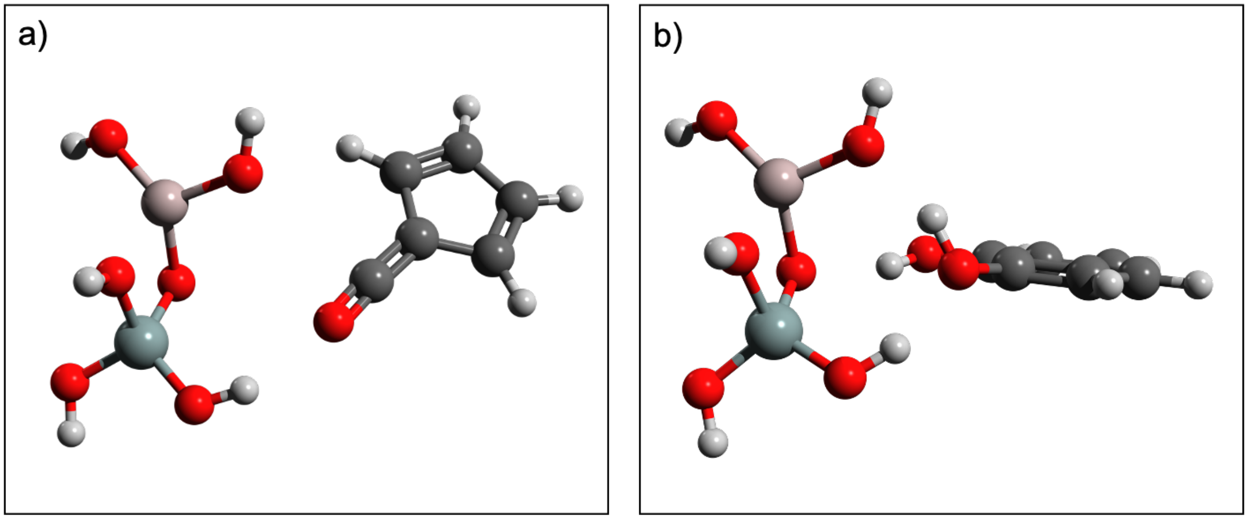

Supplementary Fig. 7 | Model of the adsorption configurations of fulvenone (a) and catechol (b) on a model catalyst $(OH)_3Si$–O–$Al(OH)_2$ at G4 level of theory obtained with Gaussian16. Fulvenone and catechol and are bound by 29 and 89 kJ/mol, respectively, in line with weak hydrogen bonding in the former. Thus, fulvenone is desorbed more easily from the catalyst than catechol.

References

1. Hemberger, P. *et al.* The Threshold Photoelectron Spectrum of Fulvenone: A Reactive Ketene Derivative in Lignin Valorization. *ChemPhysChem* **21**, 2217–2222 (2020).
2. da Silva Filho, D. A. *et al.* Vibronic coupling in the ground and excited states of the naphthalene cation. *Chem. Commun.* 1702–1703 (2004) doi:10.1039/B403828B.
3. Ennaert, T. *et al.* Conceptual Frame Rationalizing the Self-Stabilization of H-USY Zeolites in Hot Liquid Water. *ACS Catal.* **5**, 754–768 (2015).
4. Morales, I. *et al.* Induction Heating in Nanoparticle Impregnated Zeolite. *Materials* **13**, (2020).
5. Pan, Z., Bodi, A., van Bokhoven, J. A. & Hemberger, P. On the absolute photoionization cross section and threshold photoelectron spectrum of two reactive ketenes in lignin valorization: fulvenone and 2-carbonyl cyclohexadienone. *Physical Chemistry Chemical Physics* **24**, 3655–3663 (2022).
6. Hansen, N. *et al.* Identification of C5Hx Isomers in Fuel-Rich Flames by Photoionization Mass Spectrometry and Electronic Structure Calculations. *J. Phys. Chem. A* **110**, 4376–4388 (2006).
7. Kanno, N. & Tonokura, K. Vacuum Ultraviolet Photoionization Mass Spectra and Cross-Sections for Volatile Organic Compounds at 10.5 eV. *Appl Spectrosc* **61**, 896–902 (2007).
8. Savee, J. *et al.* Unimolecular isomerisation of 1, 5-hexadiyne observed by threshold photoelectron photoion coincidence spectroscopy. *Faraday Discussions* (2022).
9. Hansen, N. *et al.* Photoionization mass spectrometric studies and modeling of fuel-rich allene and propyne flames. *Proceedings of the Combustion Institute* **31**, 1157–1164 (2007).
10. Zhou, Z., Xie, M., Wang, Z. & Qi, F. Determination of absolute photoionization cross-sections of aromatics and aromatic derivatives. *Rapid Communications in Mass Spectrometry* **23**, 3994–4002 (2009).
11. Taatjes, C. A. *et al.* Products of the Benzene + O(3P) Reaction. *J. Phys. Chem. A* **114**, 3355–3370 (2010).
12. Huang, J. *et al.* Molecular Orbital Insight into the Near-Threshold Photoionization Cross Sections of Monocyclic Substituted Aromatic Compounds. *Energy Fuels* **35**, 14051–14062 (2021).
